# Creating a smoother path to scientific publication

**DOI:** 10.1002/1878-0261.70280

**Published:** 2026-05-31

**Authors:** Thomas Lemberger, Kevin M. Ryan

**Affiliations:** ^1^ EMBO Heidelberg Germany; ^2^ FEBS Press Cambridge UK; ^3^ Cancer Research UK Scotland Institute Glasgow UK; ^4^ School of Cancer Sciences University of Glasgow Glasgow UK

**Keywords:** open access, review commons, reviewed preprint, scientific editors, scientific journal, scientific society publishing

## Abstract

After years of hard work, publishing your manuscript is a critical part of the scientific process to tell others about your findings, to allow scrutiny of your results and in addition, to show your capabilities as a scientist, helping forward your career. Often, however, the process is fraught with rejection, frustration and upset. Within this short editorial, we highlight a few suggestions and initiatives that we hope will make the process of publishing easier and more efficient.

## Graphical abstract

This editorial highlights the role of Review Commons in enabling a smoother, more collaborative scientific publishing journey. It showcases the partnership between FEBS Press journals, including Molecular Oncology, and Review Commons, emphasizing transparent peer review, reduced redundancy, faster editorial decisions, and streamlined publication pathways that advance life science together. Image created using ChatGPT by OpenAI.

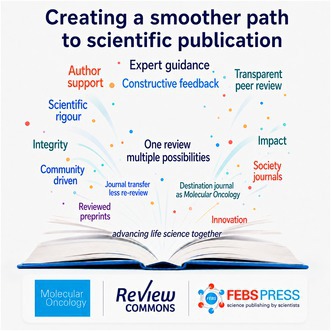

**Abbreviations**
EMBO: European Molecular Biology Organization
FEBS: Federation of European Biochemical Societies

## 1 Avoiding the pitfalls of publishing

After years of hard work, publishing your manuscript is a critical part of the scientific process to tell others about your findings, to allow scrutiny of your results and, in addition, to show your capabilities as a scientist, helping forward your career. Often, however, the process is fraught with rejection, frustration, and upset. Within this short editorial, we highlight a few suggestions and initiatives that we hope will make the process of publishing easier and more efficient.

## 2 Choosing the right journal

It is often the case that manuscripts are rejected, many times even without review, because the authors have simply chosen the wrong journal. Journals have diverse scopes and vary in their editorial selection process. In cancer biology, for example, some journals emphasize conceptual novelty in basic research, while others prioritize translational and clinical relevance. Some journals serve a wide audience, while others represent more specialized communities. It is therefore important that authors think about the best and most appropriate home for their work.

Beyond the scope of the journal—specialist or broad—here are also a few other features that authors should consider. First, is the journal a subscription journal or open access (or does it allow both options—the so‐called hybrid journals)? Open access journals make all published articles free to read from Day 1, while subscription and hybrid journals will have at least some of their published content behind a paywall. Open‐access publication also increases the visibility of your work, leading to higher citations and prestige. The choice between publication models is not simply a matter of personal view on what is deemed to be appropriate by authors; many institutions or funders also mandate open‐access publishing and sometimes even in fully open access journals.

In addition to the question of open access, authors may also wish to consider whether to publish in a society journal. Commercial journals create profit for private owners, while society journals often serve the scientific community with the revenue generated being utilized to support the society's initiatives. In the case of FEBS Press (https://febs.onlinelibrary.wiley.com/) for example, all profits from the journals owned by FEBS (including *Molecular Oncology*; https://febs.onlinelibrary.wiley.com/journal/18780261) are used by FEBS (https://www.febs.org/) to support early career researchers through fellowships, travel grants, advanced training, and scientific events. In addition, FEBS Press journals are supported by academic editors who are active researchers in the field. This ensures that your manuscript is seen by scientists who may be better equipped to understand your work and recognize its relevance if they work in your area of research.

Lastly, it is also very important to consider how a journal may serve you beyond putting your work into print. Science is getting increasingly complex, and it can be easy to make mistakes when assembling figures or referring to complicated statistics. Trustworthy journals run quality and integrity checks as part of the review process that highlight inadvertent errors, which can be corrected before publication, thereby avoiding stressful postpublication corrections or retractions and preventing further research by others based on the error. As a result, this is a very important aspect to consider.

## 3 Avoiding the scoop—Journals with antiscoop policies

It has been said many times, quite harshly, that someone's work has been ‘scooped’. What this means is that at some point during the development of a research project, another team have published similar findings, resulting in the novelty of one's own work being greatly diminished. This can be frustrating at any stage, but it can be particularly hard to accept if your experimental work is complete and your manuscript is being prepared or even being considered for publication. However, showing that findings can be independently reproduced has immense value. It serves to substantiate the previously published work and can often result in equivalent numbers of citations for your work, especially when published only a few weeks or months after the similar study. As a result, some journals, including *Molecular Oncology* have adopted ‘antiscoop’ policies whereby if similar work is published during the review of your own, this will not affect the likelihood of your work being published. This can be a very important consideration if you feel that your work is so topical that similar studies may be underway in other laboratories.

## 4 Avoiding the bounce—The principle behind review commons

A significant issue that causes delays and increases frustration in the process of publication is when manuscripts are reviewed, rejected, then reviewed again by another journal, potentially rejected a second time and so on. To avoid this issue, *Molecular Oncology* (together with the other journals of FEBS Press) has recently partnered with Review Commons (https://reviewcommons.org), which is a relatively new initiative that aims to avoid duplicated peer review efforts and the consequent delays in publishing by offering an alternative route to publication. Before engaging with a journal, authors can submit a preprint to Review Commons, which performs an in‐depth journal‐agnostic peer review of the work. Authors can then seamlessly transfer their ‘reviewed preprint’ to one of the affiliated journals, including *Molecular Oncology* and other FEBS Press journals, which will use the reviews to make an editorial decision without restarting the process. If a journal declines to publish, the reviewed preprint can be transferred again to another journal within the consortium with the same reviews. When transferred, the reviewed preprint, along with the anonymous reviews and the authors' responses to the reviewers, is made publicly available to the community immediately on bioRxiv or medRxiv.

This publishing system offers many benefits for authors. It avoids multiple rounds of redundant review, making the process more efficient. Importantly, peer review is done *before* submitting the paper to any journal. Reviewers can therefore focus on the science and evidence in the manuscript, without needing to comment on how well it fits a specific journal's editorial criteria. Moreover, young scientists often fall victim to the harsh ‘publish or perish’ culture. PhD students and postdocs nearing the end of their time often need to apply for funding and positions before their papers are published. Through the partnership between *Molecular Oncology* and Review Commons, our authors can also now expand their publishing toolset with a new instrument: the reviewed preprint. With a publicly available, reviewed preprint, you can demonstrate your research output without waiting for final journal publication. For early‐career researchers, this can be a determining factor. In this vein, EMBO has already updated its funding eligibility policies to recognize reviewed preprints, and we hope other funders will follow this example.

In conclusion, Review Commons broadens the options for publishing. After considering all the points raised in this editorial, if you are certain of the journal in which you would like to publish your work, you can of course submit directly. Alternatively, if you are less sure which journal may be interested in your work, Review Commons may well be the path forward to have your work evaluated agnostically, while still allowing publication in journals that abide by a fair, conscientious and societal approach to the dissemination of science. Moreover, if your study is about cancer and your manuscript comes to *Molecular Oncology*, either directly or via Review Commons, you can be certain that your work will be evaluated by recognized specialists in your field with a high focus on scientific quality, and that all proceedings will be used to support the scientific community.

## Conflict of interest

The authors declare no financial or personal conflicts of interest related to this editorial.

## Author contributions

TL and KR conceived and wrote the manuscript.

